# A clinically relevant and bias-controlled murine model to study acute traumatic coagulopathy

**DOI:** 10.1038/s41598-018-24225-1

**Published:** 2018-04-10

**Authors:** C. Gangloff, O. Grimault, M. Theron, K. Pichavant, H. Galinat, F. Mingant, Y. Ozier

**Affiliations:** 1 EA4324-ORPHY, Université de Bretagne Occidentale, IBSAM, UFR Sciences et Techniques, 6 Avenue Le Gorgeu, CS 93837, 29238 Brest Cedex 3, France, Brest, France; 2 Department of emergency medicine, CHU Pontchaillou, 2 Rue Henri le Guilloux, 35000 Rennes, France; 3 SAMU, SMUR and department of emergency medicine, Hôpital de la Cavale Blanche, boulevard Tanguy Prigent, Brest, France; 4 Department of biology and hemostasis, CHRU Cavale Blanche, boulevard Tanguy Prigent, Brest, France; 5 Department of anesthesiology, Hôpital de la Cavale Blanche, boulevard Tanguy Prigent, Brest, France

## Abstract

Acute traumatic coagulopathy (ATC) is an acute and endogenous mechanism triggered by the association of trauma and hemorrhage. Several animal models have been developed, but some major biases have not yet been identified. Our aim was to develop a robust and clinically relevant murine model to study this condition. Anesthetized adult Sprague Dawley rats were randomized into 4 groups: C, control; T, trauma; H, hemorrhage; TH, trauma and hemorrhage (n = 7 each). Trauma consisted of laparotomy associated with four-limb and splenic fractures. Clinical variables, ionograms, arterial and hemostasis blood tests were compared at 0 and 90 min. ATC and un-compensated shock were observed in group TH. In this group, the rise in prothrombin time and activated partial thromboplastin was 29 and 40%, respectively. Shock markers, compensation mechanisms and coagulation pathways were all consistent with human pathophysiology. The absence of confounding factors, such as trauma-related bleeding or dilution due to trans-capillary refill was verified. This ethic, cost effective and bias-controlled model reproduced the specific and endogenous mechanism of ATC and will allow to identify potential targets for therapeutics in case of trauma-related hemorrhage.

## Introduction

Every year, 5.6 million people die worldwide as a result of trauma. The four leading causes of trauma are road traffic injuries, self-inflicted violence, inter personal violence and falls, responsible for 25, 16, 10 and 6% of death, respectively. Almost 50% of them affect young people aged between 15 and 44 years, explaining their social impact and financial burden to the healthcare system^1,2^. Uncontrolled bleeding is a major cause of preventable death in this setting^3^.

Major trauma with hemorrhage is associated with a specific pathophysiologic sequence. The initial phase is characterized by a macrocirculatory failure. The effects of hypovolemia are initially compensated by an activation of the sympathetic system^4^. Tachycardia and peripheral vasoconstriction maintain blood pressure and tissue perfusion in critical organs. This state is denominated “compensated shock”. If compensation mechanisms are overwhelmed, arterial blood pressure decreases and death occurs due to the inability to maintain tissue perfusion in critical organs. This state is called “un-compensated shock”. In parallel, microcirculation regulates the distribution of blood flow within the capillary network of each organ in order to match oxygen delivery and demand and optimize oxygen extraction^4^. In a later phase, after several days, activation of the immune system leads to similar patterns such as those described in sepsis. Microcirculatory dysfunction sustains tissue hypoperfusion and multiple organ failure. This phenomenon explains the occurrence of delayed mortality despite the restoration of a normal blood pressure^5,6^.

An acute traumatic coagulopathy (ATC) dramatically increases blood losses. This specific coagulation disorder develops extremely quickly in the first 60 minutes after trauma and is detected in approximately one third of multiple injured patients upon hospital admission^7–9^. Its presence is associated with higher mortality rates and transfusion requirements^10^. ATC is an endogenous phenomenon occurring independently from external factors such as dilution, hypothermia or anti-thrombotic treatments^11^. However, external factors aggravate this pre-existing coagulopathy^12^. Therefore, in this setting, current guidelines recommend limiting fluid resuscitation, maintaining normothermia and reversing antithrombotic therapies^11^. ATC is defined as an increase in prothrombin time (PT) and/or activated partial thromboplastin time (aPTT) due to the combination of trauma and hemorrhage. The coagulation disorder is correlated with the extent of tissue trauma and the severity of shock^10^. The difficulty to characterize this condition is reflected by the variety of designations proposed, such as “acute coagulopathy of trauma shock”^13^, “disseminated intravascular coagulation with the fibrinolytic phenotype”^14^ or “trauma induced coagulopathy”^15^. ATC is a complex phenomenon, involving coagulation factors, C protein, platelets and endothelial dysfunction but its physiopathology remains unclear^16,17^. An imbalance between procoagulant and fibrinolytic pathways is suspected to be a key component of this condition during the acute phase^18^. Pro and anti-inflammatory patterns are involved in the late phase^19^.

ATC’s physiopathology and the detection of potential targets for new therapeutics are a matter of great concern for trauma-teams. Thus, the “trans-agency consortium for trauma-induced coagulopathy” (TACTICS) in USA^20^ or “task force for advanced bleeding care in trauma” in Europe focused on this topic and made proposals for further works^11,21^. In addition, international experts emphasized a lack of relevant experimental models designed to study ATC^22,23^. An ideal model should have the following characteristics^23^: low cost, mechanisms and timeline similar to real-life patients, control of exogenous factors and potential biases. Several models were developed to study ATC^22,23^ but a recent review^24^ pointed out the difficulties in studying its endogenous mechanism. Indeed, among 27 distinct animal models, only 5 triggered a coagulopathy by using the combination of trauma and hemorrhage. For example, in one of them, ATC was triggered by a tissue factor infusion without trauma. This mechanism was therefore not similar to real-life patients^25^. Some models involved high-cost protocols on pigs^26–30^. In addition, pigs seem to be in a hypercoagulable state, with C protein levels lower than in humans^31,32^. Others studies used external factors such as dilution or hypothermia to trigger coagulopathy. They were therefore not focused on the endogenous mechanism^26,27,33,34^.

In addition, a confusion factor was neither identified nor controlled in many studies^28,29,35,36^. We called it “depletion bias”. Several animal studies on ATC compare groups subjected to an hemorrhage alone with groups subjected to an hemorrhage associated with trauma^10^. This last group presented higher coagulation disorders and studies conclude to the presence of ATC. However, trauma could induce an additional bleeding in injured tissues. Therefore, higher coagulation disorders detected in traumatized animals can rather be due to this additional bleeding than to a specific mechanism linked with the combination of trauma and hemorrhage. To control this depletion bias, the amount of bleeding due to trauma has to be assessed in a specific group. Unfortunately, bleedings due to trauma cannot be easily quantified. There is no measurement tool to evaluate the amount of blood in bone or liver fractures in rats. Therefore, hemorrhage due to trauma has to be indirectly assessed using clinical and biological variables such as heart rate, blood pressure, pH, blood lactates, base excess and hemoglobin^5,15,37,38^.

The objective of this study was to develop a clinically relevant and controlled biases model of ATC to study its pathophysiology and consequences during the early phase of a major trauma.

## Materials and Methods

### Animals

Twenty-eight adult Sprague-Dawley rats (430–650 g, Janvier SAS, Le Genest St Isla, France) were housed in a controlled environment (temperature 21 ± 1 °C, relative humidity 27 ± 16%, 12–12 h light-dark cycle) with *ad libitum* access to food and water. All procedures were conducted following a protocol approved by the French ministry of agriculture (APAFIS#3797_2016012211009124) and the local university animal research ethic committee. Procedures were in accordance with the guide for the care and use of laboratory animals published by the US National Institutes of Health^39^.

### Preparation

After 1 week of housing, each rat was randomly assigned to an experimental group. Then, animals were anesthetized with an intra-peritoneal injection of ketamine (100 mg/Kg, Virbac, Carros, France) and xylazin (10 mg/Kg, Virbac, Carros, France). A temperature probe was inserted rectally and animals were placed in supine position on a warming pad (Z31SY, Ascon tecnologic, Italy) to maintain central body temperature in a normal range (37.5 ± 0.5 °C). A 2 cm cervical incision was performed, followed by a tracheostomy (2 mm diameter polyethylene tube). An arterial catheter (Leader Flex 22 G, 0,7 × 40 mm, Vygon, France) was inserted in the right carotid allowing blood sampling and continuous intra-arterial pressure monitoring. A venous catheter was inserted in the left jugular vein (Leader Flex 22 G, 0,7 × 40 mm, Vygon, France) followed by a continuous intravenous infusion of ketamin (1 mg/kg/h, Virbac inc., Carros, France). Ketamine was diluted in sodium chloride 0.9%, continuously infused at a rate of 1 ml/kg/h. Vital signs (heart rate, invasive arterial pressure and body temperature) were continuously recorded during all the procedure (MP35, Biopac systems inc. Varna, Bulgaria).

### Experimental procedure

**Figure 1:**
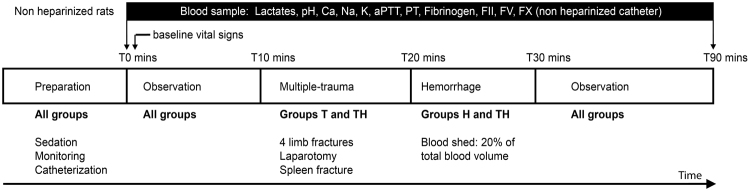
Experimental protocol. Group C, without trauma without hemorrhage; T, trauma without hemorrhage; H, hemorrhage without trauma; TH, hemorrhage with trauma (n = 7 in each group).

The experimental procedure was summarized in Fig. 1A total of 29 rats were included. One was excluded due to technical issue during blood collection (catheter misplacement). The 28 rats studied were allocated randomly to one of the 4 groups (n = 7 per group): Control (C) in which trauma and hemorrhage were not performed; Trauma (T) in which trauma was performed but not hemorrhage; Hemorrhage (H) in which hemorrhage was performed but not trauma; Trauma and hemorrhage (TH) in which trauma and hemorrhage were performed. In groups H and TH, 20% of total blood mass was gently collected in 10 min with a 10 ml syringe through the arterial catheter. Total blood mass was estimated at 5% of body weight. In groups T and TH, multiple traumas were performed as follows: 4 closed limb fractures on mid-height of the bone (2 femurs, 2 humerus) at 90 angular degrees with pliers (water pump pliers 250 mm, Facom, France). Then a 4 cm median laparotomy, and four spleen crushings of 1 cm on the inferior border of the spleen were done with surgical scissors and a needle holder, respectively (KS-463, Keysurgical, U.S.A.).

### Observation time

A very short observation time was chosen to investigate the endogenous mechanism of ATC. Indeed, transcapillary refill begins in the first minutes after hemorrhage. This compensatory mechanism is characterized by the transfer of interstitial fluid to the intravascular area, limiting the consequences of hypovolemia^40–42^. It also leads to dilution and constitutes a potential confounding factor. Thus, biological analyses at a later stage would have investigated the combined effect of both ATC and dilution coagulopathy due to transcapillary refill. This could have impaired the identification of the specific mechanisms related to ATC.

### Blood Samples

No injection of heparin was performed during the whole experimentation. Blood samples were collected through the arterial catheter. Catheter was flushed with 50 μL of 0.9% NaCl without anticoagulant after each sampling, to prevent clot formation. A 1 ml syringe rinsed with heparin 500 UI/ml was used for point of care tests. A 1.4 ml 3.2% citrated tube (Monovette, Saerstedt France) was used for hemostasis tests. Citrated tubes were gently turned upside-down 5 times. Three 15 min centrifugations were performed: one at 1000 g and two at 3000 g (centrifuge 2–16 K, Sigma, Germany) to obtain poor platelet plasma. Plasma was frozen at −80 °C until measurements.

### Blood analysis

Arterial blood pH, concentrations of hemoglobin, lactate, calcium, sodium and potassium were measured instantaneously after blood collection with a point-of care analyser (ABL80 FLEX, Radiometer, Copenhagen, Denmark). Hemostasis analysis (FII, FV, FX, fibrinogen, PT and aPTT) were performed on platelet poor thawed plasma on an automated analyser (STA-R Evolution, Stago, Asnieres sur Seine, France). PT, aPTT times and fibrinogen concentrations were measured with specific reagents, « neoplastine Cl + 10 », « triniclot aPTTb », and « STA liquid fib », respectively. Specific factor-depleted plasmas (Stago, Asnieres sur Seine, France) were used to determine coagulation factor times. Coagulation factor activities were expressed in time-to-clot instead of percentages in this study. Indeed, these percentages are expressed in comparison with the laboratory reference in humans and references for rats are not available. Therefore, we made the choice to express non-modified data: the time to clot in seconds. This time is inversely proportional to the factor activity.

### Statistical analysis

Statistical analyses were performed with “SPSS statistics for Macintosh” software version 21 (I.B.M. corp., Armmonk, NY, 2012). Line graphs, boxplots and histograms were performed with “Prism 7 for Mac OS X” version 7.0a (Graphpad software, La Jolla, USA, 2016). Considering that the PT and aPTT values obtained at 0 min are reference values for each animal, ratios were calculated as follows: PT ratio = (PT at 90 min)/(PT at 0 min), aPTT ratio = (aPTT at 90 min)/(aPTT at 0 min). Two-way repeated measures analyses of variance with adequate post hoc tests were used to compare means over time within and between groups. Results were expressed as the mean ± standard error of mean (SEM), unless otherwise stated. A p-value of <0.05 was considered statistically significant.

### Availability of data and material

The datasets used and/or analyzed during the current study are available from the corresponding author on reasonable request.

## Results

### Group comparability and effects of anesthesia

**Figure 2:**
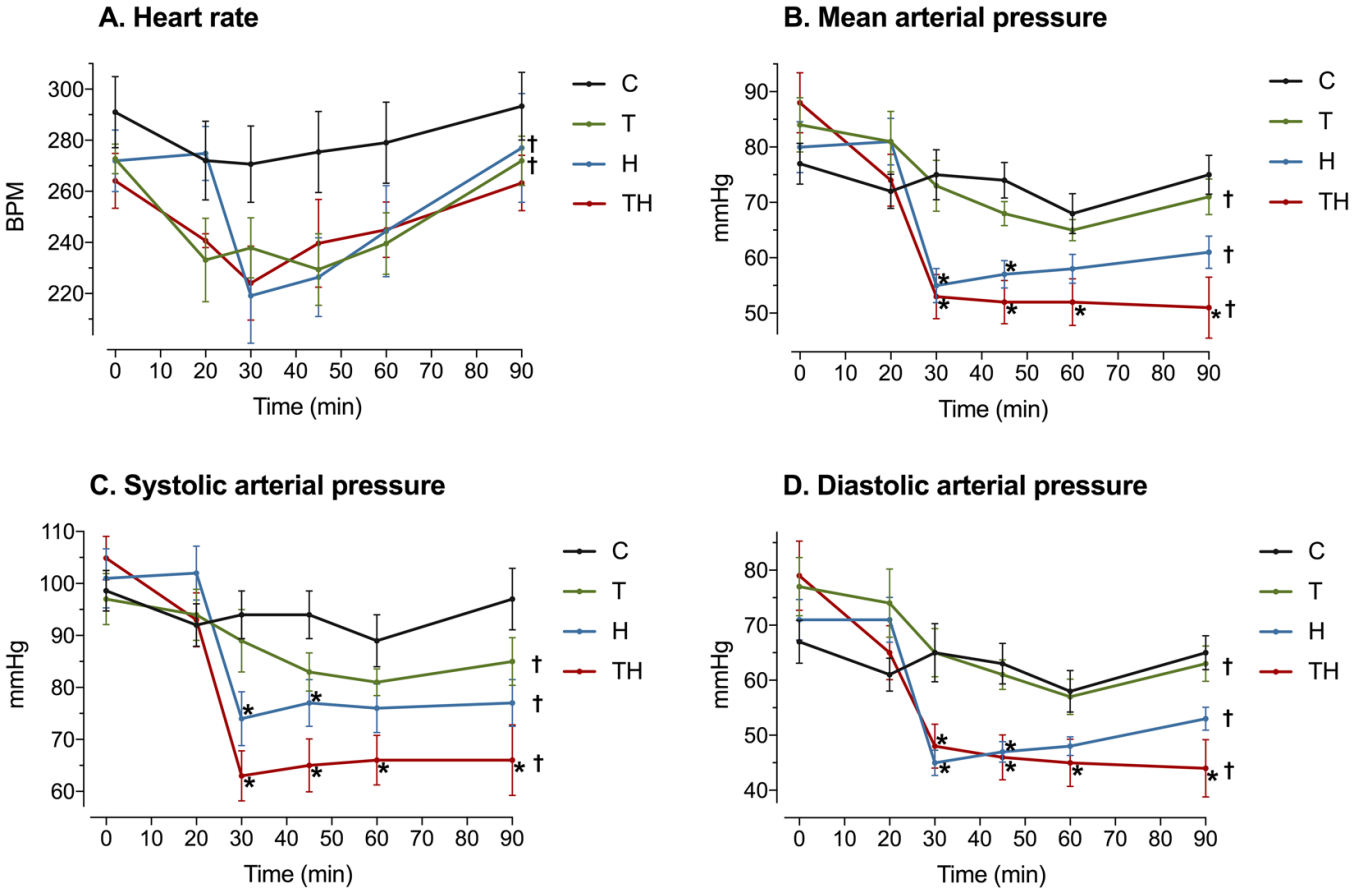
Hemodynamic variables. C, Control; T, Trauma; H, Hemorrhage; TH, Trauma and hemorrhage, n = 7 per group. Values represent mean ± SEM. *p < 0.05 compared with group C; ^†^p < 0.05 for repeated measure ANOVA, meaning a statistically significant effect of time in concerned group.

At 0 min, no statistical difference between groups was observed for all variables (p > 0.05). In addition, anesthetic agents did not interfere with hemodynamic because heart rate and MAP did not vary significantly over time in group C (p > 0.05, Fig. 2A,B). These results allow comparisons between groups at the end of the experimentation without doubt concerning potential bias due to the procedure.

### ATC was reproduced in this model

**Figure 3:**
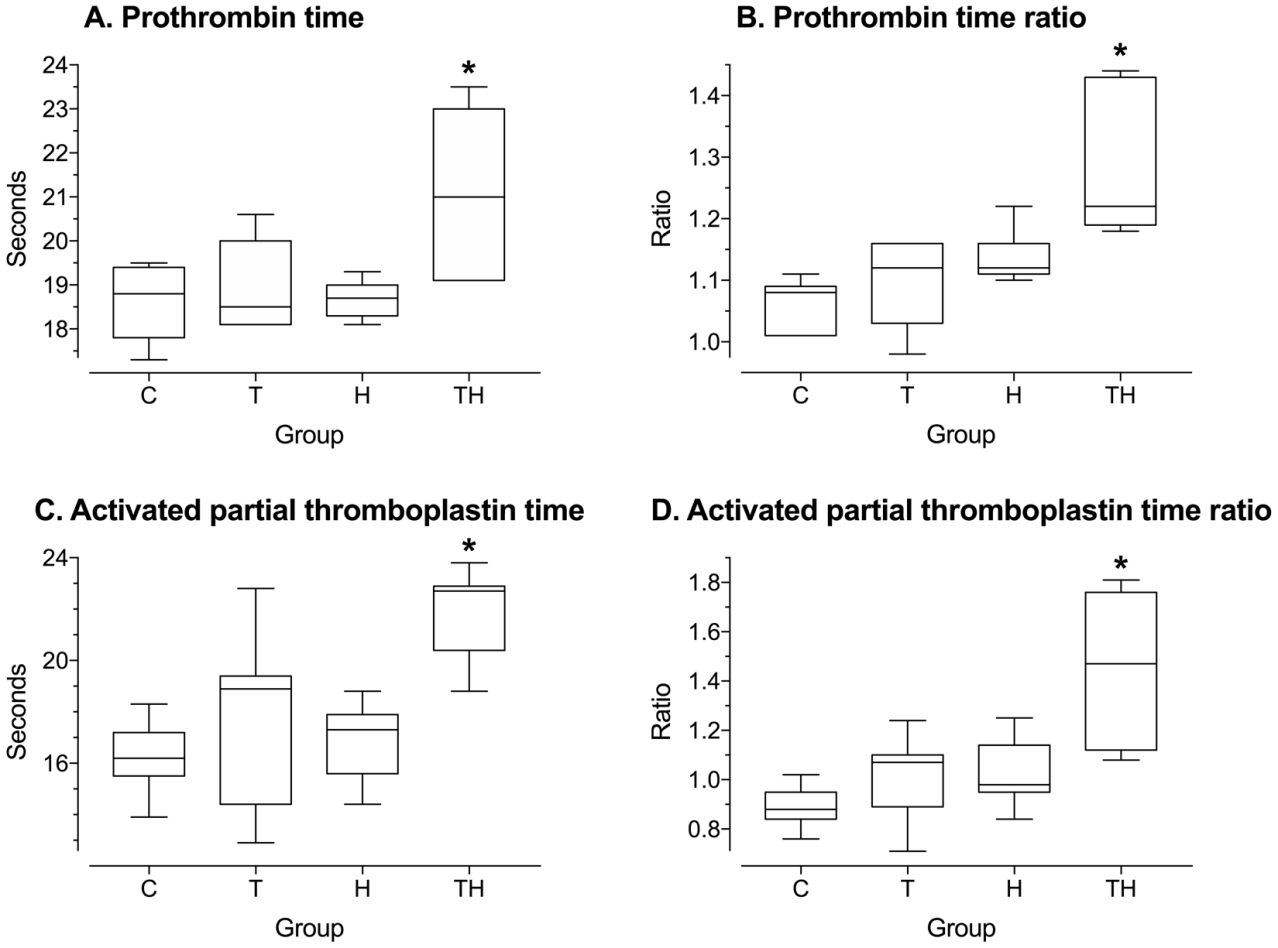
General coagulation assays. Box and whisker plot of general coagulation assays at 90 min in each experimental group. C, Control; T, Trauma; H, Hemorrhage; TH, Trauma and hemorrhage, n = 7 per group. Whiskers represent the 5th and 95th percentiles. *p < 0.05 versus C T and H. Ratios were calculated for each animal as follows: (value at 90 min)/(value at 0 min).

The main objective of this study was to reproduce the specific mechanism leading to ATC. However, trauma or hemorrhage alone could induce coagulation impairments. Thus, we studied their single effects on coagulation in group T and H, and compared them to their association in group TH. Mean PT, PTr, aPTT and aPTTr increased significantly in group TH compared to group C, T and H (p < 0.05, Fig. 3A–D). The four main biological markers of ATC were therefore present in the TH group^10,11^. This coagulation disorder was specifically induced by the association of trauma and hemorrhage because no statistical difference between C, T and H groups was retrieved for these variables (p > 0.05, Fig. 3A–D). The absence of potential confounding factors that may interfere with coagulation was also verified. No significant decrease in hemoglobin was observed in group H, suggesting the absence of transcapillary refill^40^. As mentioned in the “observation time” section, this compensatory mechanism leads to dilution and could have interfered with ATC. In addition, we neither used fluid replacement nor antithrombotic injection that could dilute coagulation factors or modify their activities. We also prevented hypothermia which is known to reduce clotting factor enzymatic protease activity *in vivo*. Coagulopathy was therefore due to a specific and endogenous mechanism, strictly corresponding to the definition of ATC.

### Un-compensated shock was triggered by ATC

**Figure 4:**
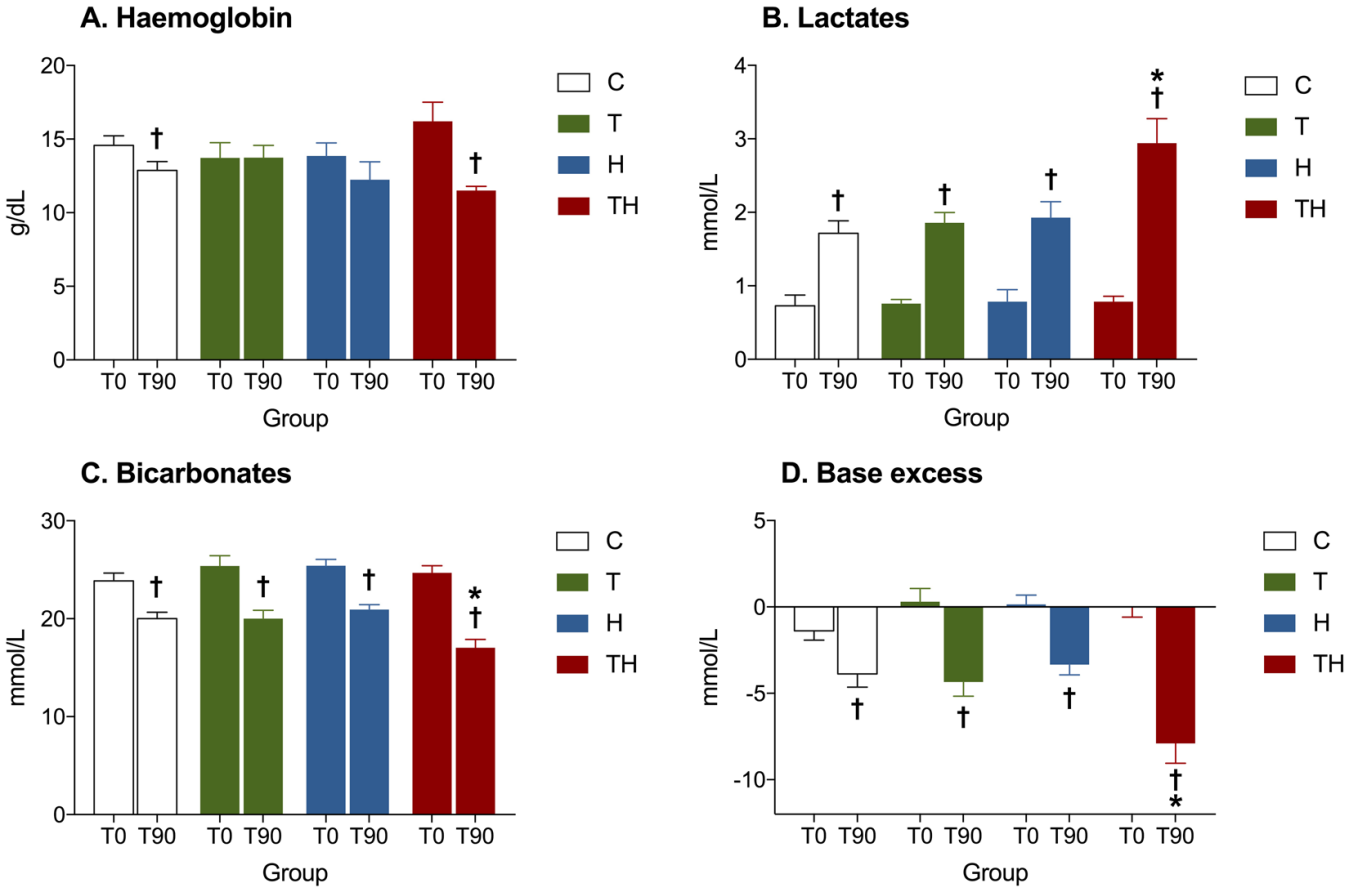
Shock variables. Mean values at 0 min and 90 min in each experimental group. C, Control; T, Trauma; H, Hemorrhage; TH, Trauma and hemorrhage, n = 7 per group. ^†^p < 0.05 compared with 0 min in the same group, *p < 0.05 versus C T and H. Data are presented as mean ± SEM.

Next, we wanted to evaluate the clinico-biological consequences of ATC, which is a key point to explain higher mortality rates in case of trauma-related hemorrhage. In group TH, we observed a significant decrease in base excess concentrations (p < 0.05, Fig. 4D) associated with an increase in lactate (p < 0.05, Fig. 4B) and potassium concentrations (p < 0.05, Table 1). The rise in lactate concentration evidences the presence of cellular hypoxemia and the transition from aerobia to anaerobia. The lowering in base excess reflects a metabolic acidosis and the rise in potassium concentration indicates the presence of cellular leakage. These results indicate an energetic imbalance between cellular incomes and needs leading to cellular dysfunction, corresponding to the definition of shock: “a state in which the circulation is unable to deliver sufficient oxygen to meet the demands of the tissues”^43^. In addition, arterial pressure remained low until the end of the experimentation in group TH (p < 0.05, Fig. 2B–D). Compensatory mechanisms were therefore not sufficient to maintain blood pressure; this clinico-biological status is also known as un-compensated shock. In contrast, arterial pressures felt just after trauma/hemorrhage and raised slowly during the experimentation in group T and H (p > 0.05, Fig. 2B–D). Biological markers of shock were also negative in these groups (p > 0.05, Fig. 4B–D). Consequently, ATC induced a switch from compensated to un-compensated shock in this model.

**Table 1:** Arterial blood tests and ionogram assays.

	Group C	Group T	Group H	Group TH	p-value
**Arterial blood tests**					
pH					
T0	7.35 ± 0.018	7.37 ± 0.016	7.36 ± 0.012	7.38 ± 0.005	0.441
T90	7.38 ± 0.017	7.36 ± 0.006	7.37 ± 0.017	7.32 ± 0.033	0.412
pO2 (mmHg)					
T0	69.2 ± 3.3	62 ± 2.5	66 ± 1.8	69.8 ± 2.5	0.158
T90	83.8 ± 6.6	79.2 ± 2	71.8 ± 4.9	77.2 ± 5.6	0.437
pCO2 (mmHg)					
T0	44.8 ± 3.3	44.5 ± 2.5	46 ± 1.8	42.4 ± 2.5	0.748
T90	34.43 ± 1.6^†^	36.1 ± 1.1^†^	36.9 ± 1.8^†^	34.5 ± 2.8^†^	0.760
**Ionogram**					
Calcium (mg/L)					
0 min	1.36 ± 0.46	1.42 ± 0.02	1.33 ± 0.02	1.29 ± 0.03	0.186
90 min	1.38 ± 0.03	1.25 ± 0.02^†^	1.3 ± 0.04	1.4 ± 0.11	0.336
Sodium (mEq/L)					
0 min	140 ± 1.49	142 ± 0.72	141 ± 1	141 ± 1.01	0.740
90 min	143 ± 0.77^†^	144 ± 2.79	136 ± 3.5	142 ± 3.1	0.202
Potassium (mmol/L)					
0 min	5.34 ± 0.24	5.14 ± 0.34	5.09 ± 0.13	5.57 ± 0.23	0.527
90 min	5.39 ± 0.17	6.03 ± 0.26	5.80 ± 0.24^†^	6.73 ± 0.26^a^	0.005*

Data are presented as mean ± SEM. C, Control; T, Trauma; H, Hemorrhage; TH, Trauma and hemorrhage, n = 7 per group. ^a^p < 0.05 compared with C, ^†^p < 0.05 compared with 0 min in the same group.

### ATC was associated with a specific coagulation profile

**Figure 5:**
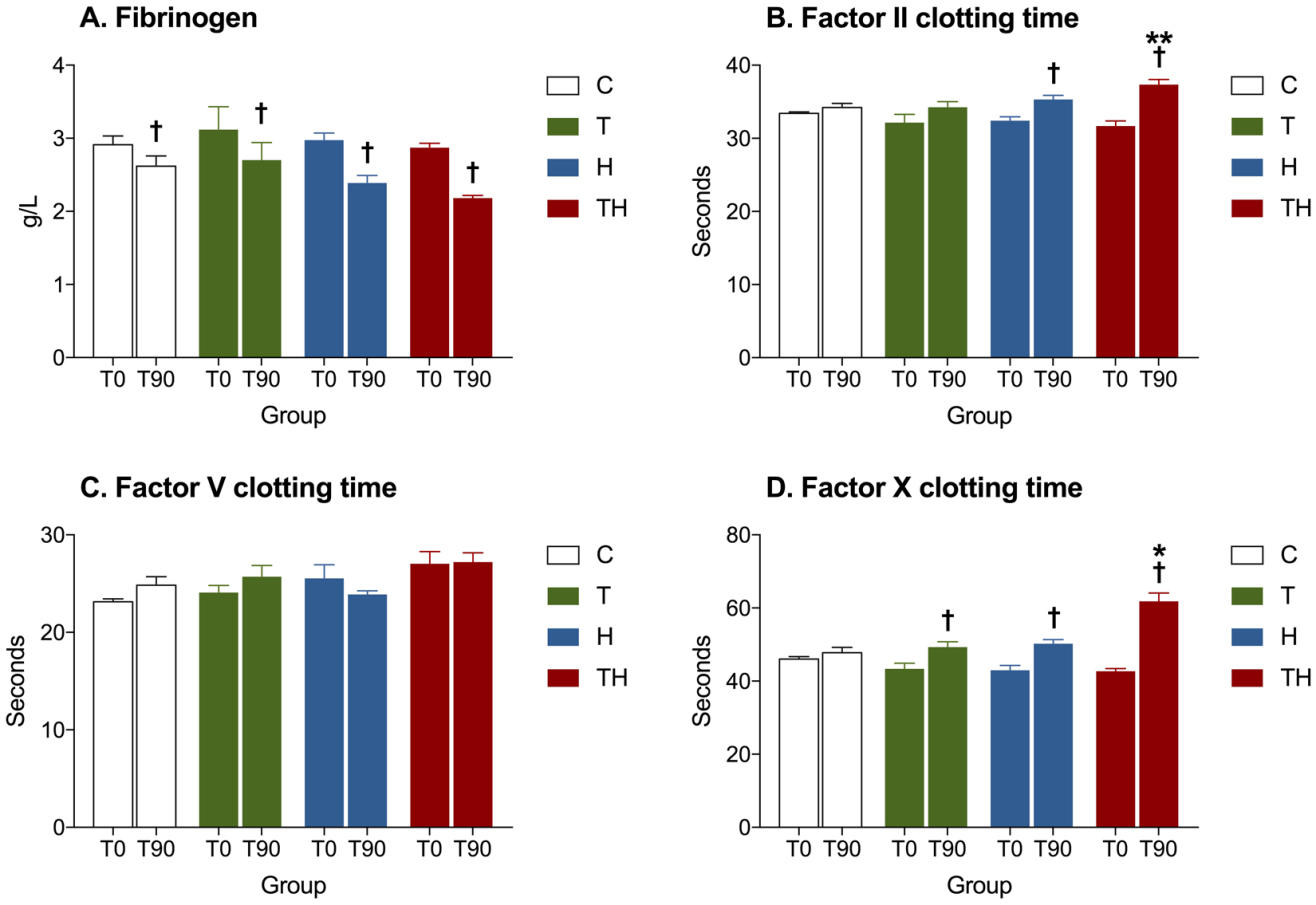
Coagulation factors. Histograms of specific coagulation factors at 0 min and 90 min in each experimental group. C, Control; T, Trauma; H, Hemorrhage; TH, Trauma and hemorrhage, n = 7 per group. ^†^p < 0.05 compared with 0 min in the same group, *p < 0.05 versus C T and H. **p < 0.05 versus C and T. Data are presented as mean ± SEM.

We then studied specific coagulation factors to identify their role in case of ATC. A significant decrease in fibrinogen concentration was observed in all groups, probably due to clot formation after cervical incision and cannulation (Fig. 5A). Interestingly, a significant negative linear relationship between PT ratio and fibrinogen serum concentrations at 90 min was retrieved (intercept, 3.57; coefficient, −1.04; p, 0.0272; R^2^, 0.6561; adjusted R^2^, 0.5873). This result highlights a protective effect of fibrinogen against ATC. In addition, a significant increase of FII and FX times from 0 to 90 min was observed in this study (p < 0.05, Fig. 5B–D). These data suggest that specific coagulations pathways mediated by FII and FX are involved in ATC’s genesis.

## Discussion

This model was particularly consistent with human ATC in terms of temporality, type of injuries, compensation mechanisms and coagulation impairments. Indeed, in most countries, patients are brought to hospital in approximately 60 min^44^ and injuries performed in this study were consistent with those retrieved after traffic accidents^44^. In addition, compensatory mechanisms were similar to those described in humans to maintain blood pressure and homeostasis. Concerning blood pressure, we observed a decrease in heart rate in group T, H ad TH, suggesting an activation of the Bezold Jarisch reflex (Fig. 2A). This reflex increases end-diastolic volumes and cardiac output by lengthening the time allowed to fill cardiac chambers^45–47^. It is triggered by left ventricle stretch receptors, endogenous catecholamins and serotonin. Serotonin is released after platelet activation, explaining bradycardia in T and TH groups. Stretch receptor and catecholamins are activated in case of hypovolemia, explaining bradycardia in H and TH groups. Concerning homeostasis, alveolar hyperventilation and bicarbonates consumption to maintain pH in normal range were retrieved in this model (Table 1). Moreover, the protective role of fibrinogen was observed^48^ and values for aPTT and PT ratios were comparable to those retrieved in humans when ATC occurs^10^.

The role of previously identified confounding factors was then assessed to guarantee the robustness of the model. The absence of transcapillary refill was established by comparing hemoglobin concentrations at 0 and 90 min in the H group to ensure the absence of spontaneous dilution due to hemorrhage (Fig. 4A). The “depletion bias” was assessed by comparing markers of hemorrhage to ensure the absence of significant bleeding in the trauma group. In our model, mean arterial pressure, base excess, hemoglobin, bicarbonates and lactate concentrations were similar in group C and T at the end of the experimentation and did not vary during time in group T (Figs 2B and 4A–D). Therefore, trauma alone did not lead to any significant bleeding. As far as we know, this study is the first to identify and verify the absence of these two major bias.

Then, we wanted to illustrate the usefulness of our model to explore ATC’s pathophysiology. To this purpose, analysis of coagulation factors concentrations were performed and allowed us to hypothesize that TFPI could play a role in case of trauma-related hemorrhage. Indeed, activation of C-protein pathway is known to be one of the key drivers of ATC^49–51^. This activation leads to a decrease in FV^52,53^. Interestingly, in our study, ATC occurred in group TH without any change in FV time. On the contrary, FX time raised, indicating a decrease of FX activity. These data suggest that APC’s activation is not the only mechanism to trigger ATC. Another pathway inhibiting specifically FX appears to be involved in this context. A valid theory could be that FX was repressed by an inappropriate secretion of tissue factor pathway inhibitor (TFPI). TFPI is a serine protease inhibitor^54^. One of its major isoform, TFPIα, is localized on endothelium and platelets in humans, and only in platelet in murines^55^. It is externalized after platelet activation and has 3 Kunitz domains^56–58^. The first domain binds to the active site of FVIIa, the second to the active site of FX and the third to protein S^59,60^. This structure explain why TFPI induces a specific repression of FX^61–63^. Consequently, an increase in TFPI plasma concentration could explain the inhibition of FX observed in this experimentation. These findings reinforce the theory that platelets and/or endothelium play a role in ATC’s genesis.

In addition, our model has particularities that could be of interest for further studies. As far as we know, it is the first to demonstrate and reproduce the switch from compensated to un-compensated shock due to ATC. This makes possible to explain why higher mortality rates are observed in this condition. Moreover, it is the first to show that ATC can arise even in case of moderate hemorrhage. Indeed, ATC occurred with only a loss of 20% of total blood mass whereas 40% were usually necessary in previous studies. In addition, no animal died before the end of the procedure. This small mammalian model is therefore ethic, time and cost-effective.

The main limitation of this model is that average total blood mass of rats is about 25 ml. Volumes were therefore limited to avoid hemodynamic disturbance due to blood samples. High volumes or repeated blood samples, for example to investigate the time course of multiple coagulation factors in the same time, are therefore excluded. This constraint applies to all small mammalian models. In further studies we should consider that one general assay such as PT or aPTT will be sufficient to conclude to the presence of ATC. The remaining blood will be available to explore ATC’s pathophysiology. Others limitations should be considered: Clotting factors serum concentrations are not similar among species; quantitative results are therefore not strictly transferable to humans. However, the role of coagulation in survival is so crucial that key mechanisms were probably conserved between species during evolution.

In conclusion, this model reproduced the sequence and timeline retrieved in humans in case of severe trauma associated with hemorrhage. The combination of these two factors induced a specific and endogenous coagulopathy fitting the definition of ATC. A complete consistence with humans was observed concerning clinical and biological markers for shock, compensation mechanism, protective factors and coagulation disorders involved in this condition. We identified and controlled two new bias: majored bleedings due to trauma and dilution due to transcapillary refill. Moreover, we reproduced the switch from compensated to un-compensated shock when ATC occurs, corresponding to the time when compensation mechanisms are overwhelmed. This could help to understand the pathophysiology of ATC at this key moment and to identify potential targets for therapeutics to decrease mortality. In addition, we demonstrated that ATC can occur even in case of moderate hemorrhage and we made the hypothesis that TFPI could repress FX in this case. This model is therefore effective to explore coagulation patterns involved in the genesis of ATC and its consequences on tissue perfusion.
